# Supplementary material for: Towards Meaningful Interpretation of Molecular Data: Insights Gained from HMMD Challenges in Salmonella Detection for Future NGS Integration in Clinical Microbiology
Source: Diagnostics (Basel). 2024 Dec 31;15(1):77. doi: 10.3390/diagnostics15010077 (PMC11720086; doi:10.3390/diagnostics15010077)
Supplement: Supplementary file 1 [file diagnostics-15-00077-s001.zip › diagnostics-3331567-supplementary.pdf]

**Supplemental Table S1.** Comparison of FDA-approved 'Gastrointestinal Pathogen Panel Multiplex Nucleic Acid-Based Assay Systems.

| Device Name | **BIOFIRE FILMARRAY Gastrointestinal Panal** | **VERIGENE Enteric Pathogen Nucleic acid test** | **xTAG Gastrointestinal Pathogen Panel** | **BD MAX Extended Enteric Bacterial Panel** | **QIAstat-Dx Gastrointestinal Panel 2** | **Biocode Gastrointestinal Pathogen Panel** |
|---|---|---|---|---|---|---|
| Company | BioFire Diagnostics, LLC | Nanosphere, Inc. | Luminex Molecular Diagnostics, Inc. | Becton, Dickinson and Company | QIAGEN Gmbh | Applied Biocode, Inc. |
| The First FDA Approval Date | May 02, 2014 | Jun 20, 2014 | Mar 21, 2013 | May 02, 2017 | May 31, 2024 | Sep 28, 2018 |
| DNA Amplification | PCR | PCR | PCR | PCR | PCR | PCR |
| Time to Result | ~ 1 hour | ~ 2 hours | 5 hours | ~ 3 hours | ~ 1 hour | < 5 hours |
| Specimen Type | Stool in Cary Blair transport media | Stool in Cary Blair transport media | Unpreserved Stool | Unpreserved stool or Cary-Blair preserved stool | Preserved stool in Para-Pak C&S or Fecal Swab transport media | Unpreserved stool or Cary-Blair preserved stool |
| Detection Method | Nested multiplex PCR followed by high resolution melting analysis to confirm the identity of amplified product | Gold/Silver nanoparticle probe detection of bacterial-specific DNA on complementary oligo- microarray | Specific microbial target or control bead populations coupled to sequences from Universal Array, streptavidin, phycoerythrin conjugate | Real time, multiplex PCR using Fluorogenic target-specific hybridization | Real time, multiplex PCR using Fluorogenic target-specific hybridization | Multiplex RT-PCR and probe hybridization followed by fluorescence detection and decoding of barcoded magnetic beads (BMB) that are coupled to biotinylated products with streptavidin conjugate |
| Number of Detected Targets | 22 | 9 | 15 | 6 | 16 | 18 |

| | | (13 bacteria, 5 viruses, and 4 parasites) | (5 bacteria, 2 viruses, and 2 toxins) | (9 bacteria, 3 viruses, and 3 parasites) | (4 bacteria, and 2 toxins) | (8 bacteria, 4 viruses, and 4 parasites) | (12 bacteria, 3 viruses, and 3 parasites) |
|---|---|---|---|---|---|---|---|
| Bacteria | Campylobacter spp. | + (C. jejuni, C. coli, C. upsaliensis) | + (C. jejuni, C. coli, and C. lari) | + (C. jejuni, C. coli and C. lari) | + (C. jejuni, and C. coli) | + (C. jejuni, C. coli and C. upsaliensis) | + (C. jejuni, C. coli) |
| | Clostridium difficile toxin A/B | + | N/A | + | N/A | N/A | + |
| | EAEC | + | N/A | N/A | N/A | N/A | + |
| | EPEC | + | N/A | N/A | N/A | + (Not reported for fecal swab) | N/A |
| | ETEC | + | N/A | + | N/A | + | + |
| | STEC | + | N/A | + | N/A | + (Not reported for fecal swab) | + |
| | E.coli O157 | + | N/A | + | N/A | | + |
| | EIEC | + | N/A | | + | + | + |
| | Shigella spp. | + (Plesiomonas shigelloides only) | + (S. dysenteriae, S. boydii, S. sonnei, and S.flexneri) | + (S. boydii, S. sonnei, S. flexneri and S. dysenteriae) | + | + (Plesiomonas shigelloides only) | + |
| | Salmonella spp. | + | + | + | + | + | + |
| | Vibrio parahaemolyticus | + | + | N/A | N/A | N/A | + |
| | Vibrio spp. | + (Vibrio cholerae only) | + (V.cholerae only) | + (Vibrio cholerae only) | N/A | N/A | + |
| | Yersinia enterocolitica | + | + | + | N/A | + | + |
| Virus | Adenovirus 40/41 | + | N/A | + | N/A | + | + |
| | Astrovirus | + | N/A | | N/A | + | N/A |
| | Norovirus GI/GII | + | + | + | N/A | + | + |
| | Rotavirus A | + | + | + | N/A | + | + |

| | | | | | | | |
|---|---|---|---|---|---|---|---|
| | Sapovirus | + | N/A | | N/A | N/A | N/A |
| Parasite | Cryptosporidium spp. | + | N/A | + (C. parvum and C. hominis only) | N/A | + | + (C. parvum/C. hominis) |
| | Entamoeba histolytica | + | N/A | + | N/A | + | + |
| | Giardia lamblia/intestinalis | + (G. intestinalis and G. duodenalis) | N/A | + (G. lamblia only - also known as G. intestinalis and G. duodenalis) | N/A | + (G. lamblia only - also known as G. intestinalis and G. duodenalis) | + |
| | Cyclospora cayetanensis | + | N/A | N/A | N/A | + | N/A |
| Toxin | stx1/stx2 | N/A | + | N/A | + | N/A | N/A |

Abbreviations: FDA, Food and Drug Administration; PCR, Polymoerase Chain Reaction; EAEC, Enteroaggregative Escherichia coli; EPEC, Enteropathogenic Escherichia coli;

ETEC, Enterotoxigenic Escherichia coli; STEC, Shiga-like toxin-producing Escherichia coli; EIEC, Enteroinvasive Escherichia coli; N/A, Not Applicable.
